# A huge cardiac haemangioma in the left ventricular wall

**DOI:** 10.1093/ehjcr/ytaa374

**Published:** 2020-11-14

**Authors:** Yutaro Miyoshi, Takeshi Kitai, Takafumi Yamane, Madoka Sano, Tadaaki Koyama, Yutaka Furukawa

**Affiliations:** 1 Department of Cardiovascular Medicine, Kobe City Medical Center General Hospital, 2-1-1 Minatojima-minamimachi, Chuo-ku, Kobe 6500047, Japan; 2 Department of Cardiovascular Medicine, Nishinomiya Watanabe Cardiovascular Center, Nishinomiya, Japan; 3 Department of Cardiothoracic Surgery, Kobe City Medical Center General Hospital, Kobe, Japan

A 76-year-old asymptomatic male patient was referred to the cardiology department because of an abnormal electrocardiogram (ECG) finding, namely giant negative T waves, mimicking apical hypertrophic cardiomyopathy (*Panel A*). There were no significant abnormal findings on physical examination or laboratory testing. Transthoracic and transoesophageal echocardiography demonstrated a large round mass (47 mm × 50 mm) in the anterolateral left ventricular free wall (*Panel B*). Contrast-enhanced computed tomography (CT) scans revealed spotty calcification and heterogeneous contrast enhancement within the tumour (*Panel C*). Coronary angiography indicated contrast pooling in the tumour from the diagonal branch, the so-called tumour blush (*Panel D*). Coronary CT angiography clearly showed that the diagonal branch of the coronary artery was embedded in the tumour (*Panel E*). In magnetic resonance imaging, the mass had high-intensity signals in T2-weighted imaging (*Panel F*). Positron emission computed tomography showed mild hypermetabolism of the left ventricle [maximal value of standardized uptake value (SUV_max_): 4.84], that was not sufficient to exclude the possibility of malignancy (*Panel G*). An open-chest needle biopsy with a minimal incision thoracotomy was performed (*Panel H*). A pathological examination verified that the mass was composed of CD31/34 positive proliferative endothelial cells lining the capillaries (*Panels I* and *J*). The mass was diagnosed as a mixed capillary and cavernous type haemangioma. Since the tumour was benign with no apparent haemodynamic concerns and the patient was asymptomatic, resection surgery or further additional treatment such as steroid therapy was not selected and regular imaging follow-up was planned.

**Figure:**
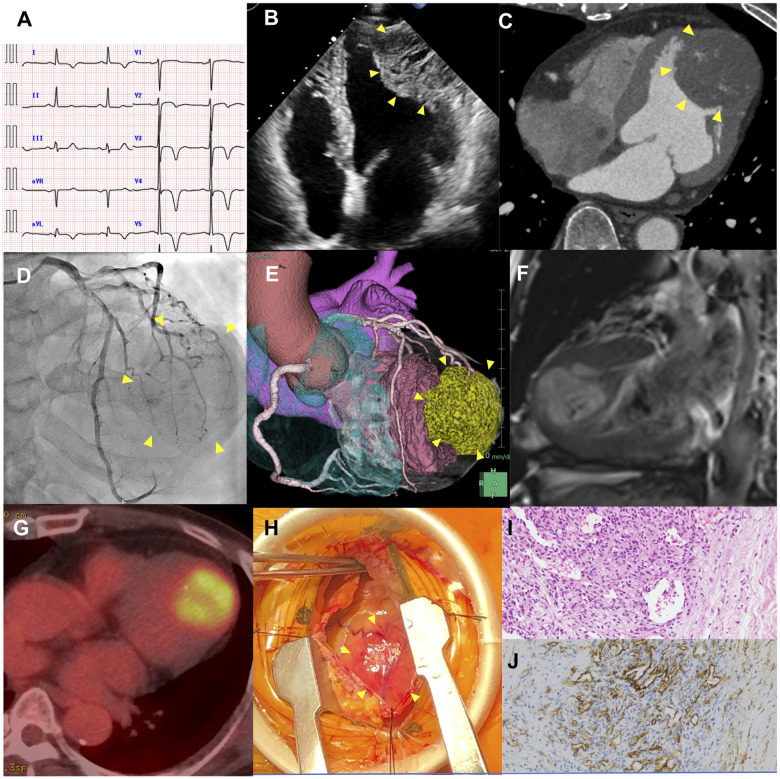


One year after the diagnosis, the patient is currently asymptomatic and the haemangioma has not increased in size.

(*Panel A*) Electrocardiogram showing giant negative T waves in I/aVL/V2–V6. (*Panel B*) Transthoracic echocardiography showing a large intramyocardial mass (about 47 mm × 50 mm) of the left ventricle. (*Panel C*) Electrocardiogram-gated contrast-enhanced computed tomography scan shows that the margins of the mass were well defined. (*Panel D*) Coronary angiography shows the mass was mainly fed by the first diagonal branch. (*Panel E*) Coronary CT angiography reconstructed image shows that the tumour was located in the anterolateral part of the left ventricle. (*Panel F*) Magnetic resonance imaging shows the mass had high-intensity signals in the T2-weighted image. (*Panel G*) Positron emission tomography showing mild hypermetabolism of the left ventricle, and no systemic metastasis. (*Panel H*) The direct view at the surgery. The tumour was elastic hard and the margin from normal left ventricular muscle tissue was relatively clear. (*Panels I* and *J*) Histological examination showed that the mass was composed of capillaries and short spindle cells in the stroma, and CD31/34 positive proliferative endothelial cells lining capillaries.

